# Bone marrow niches in myelodysplastic syndromes

**DOI:** 10.20517/2394-4722.2021.120

**Published:** 2021-07-14

**Authors:** Giovanna Tosato, Jing-Xin Feng, Hidetaka Ohnuki, Minji Sim

**Affiliations:** Laboratory of Cellular Oncology, National Cancer Institute, National Institutes of Health, Bethesda, Maryland 20892, USA.

**Keywords:** Hematological malignancies, endothelial cells, stromal cells, bone marrow niches, microenvironment, angiogenesis, inflammation, hypoxia

## Abstract

Genetic and epigenetic lesions within hematopoietic cell populations drive diverse hematological malignancies. Myelodysplastic syndromes (MDS) are a group of myeloid neoplasms affecting the hematopoietic stem cells characterized by recurrent genetic abnormalities, myelodysplasia (a pathological definition of abnormal bone marrow structure), ineffective hematopoiesis resulting in blood cytopenia, and a propensity to evolve into acute myelogenous leukemia. Although there is evidence that the accumulation of a set of genetic mutations is an essential event in MDS, there is an increased appreciation of the contribution of specific microenvironments, niches, in the pathogenesis of MDS and response to treatment. In physiologic hematopoiesis, niches are critical functional units that maintain hematopoietic stem and progenitor cells and regulate their maturation into mature blood cells. In MDS and other hematological malignancies, altered bone marrow niches can promote the survival and expansion of mutant hematopoietic clones and provide a shield from therapy. In this review, we focus on our understanding of the composition and function of hematopoietic niches and their role in the evolution of myeloid malignancies, with an emphasis on MDS.

## INTRODUCTION

Myelodysplastic syndromes (MDS) are characterized by the presence of persistent cytopenia involving one or more hematopoietic cell lineages associated with morphologic evidence of bone marrow dysplasia affecting at least 10% of bone marrow cells belonging to one or more than one cell lineages. Based on the combination of morphologic cell abnormalities and cytogenetic abnormalities, the 2016 revised classification of the World Health Organization has distinguished six MDS subtypes: MDS with a single-lineage dysplasia, MDS with multilineage dysplasia, MDS with ring sideroblasts and single-lineage dysplasia or multilineage dysplasia, MDS with isolated deletion of the chromosome 5q [del(5q)], MDS with excess blasts type 1 or type 2, and unclassifiable MDS. Myelodysplastic-myeloproliferative neoplasms (MPN) are a group of myeloid neoplasms with clinical, laboratory, and morphologic features that overlap those of MDS^[1]^.

In health, hematopoiesis relies on interactions between the hematopoietic cells and surrounding non-hematopoietic “stromal” cells for survival and function. These interactions occur in certain microenvironments, called niches, through cell-to-cell association, vesicular particles, and soluble mediators acting systemically or at short-distance. The self-renewing hematopoietic stem cells (HSC), at the top of the hematopoietic hierarchy, are dependent on their niches for survival. Mutant hematopoietic cells in MDS, like normal HSC that have self-renewing potential, can also rely on niche factors for their survival and expansion. In some cases, niches can also promote MDS resistance to treatment.

Recent advances in single-cell genomics have provided an unprecedented appreciation of the diversity of the non-hematopoietic cell populations that reside in the bone marrow and have permitted to infer functional capabilities of hematopoietic cell niches. These advances, combined with a better understanding of genetic lesions in MDS, provide an opportunity for outlining potential roles of niches in pathologic hematopoiesis. Despite improved understanding of the pathogenesis of MDS, therapeutic advances have been limited since 2005 when FDA approved lenalinomide for patients with low or intermediate risk MDS with chromosome 5q deletion. Bone marrow transplantation remains the only curative treatment for some patients. Therefore, abnormal niches provide an opportunity for discovery of new treatments. Here, we summarize our understanding of the bone marrow niches in health and their abnormalities in myeloid malignancies, particularly in MDS. This seems an important step towards development of therapies targeting the niche rather than the mutant hematopoietic cells.

## BONE MARROW NICHES IN HEALTH

Endothelial cells, mesenchymal lineage cells, and sympathetic neuronal cells are the major non-hematopoietic cell components of the normal adult bone marrow in mice and humans. Studies in the mouse have concluded that most HSC reside in proximity to the blood vessels. Consistently, the HSC niches have been provisionally defined based on their relationship of the vessels and more broadly on their anatomical location within the bone. The inner core of the bone marrow (the central niche) comprises > 90% of the bone marrow volume and contains 85% of HSC, whereas the endosteal niche at the periphery of the bone marrow near the bone harbors the remaining 15% HSC^[2,3]^. Thus, the endosteal niche is relatively enriched with HSC.

Sinusoids, arterioles, and vessels that connect sinusoids and arterioles are the types of blood vessels identified morphologically in the bone marrow [Table 1]. Sinusoids are the most abundant vascular system, accounting for 30% ± 5% of the bone marrow volume. These vessels form a highly branched and fenestrated capillary network where the individual vessels are regularly spaced by 46 ± 1 μm and are distributed throughout the length of the long bones^[4]^. Arterioles, which comprise only 1.2% ± 0.1% of bone marrow volume, are mostly present near the bone where they are fed by arteries penetrating the bone cortex^[4]^. Arterioles and sinusoids are connected by transition zone capillaries that are in the endosteal niche, mostly at the bone metaphysis^[5]^. Based on high-level expression of the surface markers endomucin and PECAM1/CD31, these transitional zone capillaries were labeled “type H” vessels, distinguishing them from sinusoidal vessels, “type L” vessels, with lower surface expression of these two markers^[5]^. Sinusoidal vessels and arteriolar/arterial vessels in the bone marrow share the endothelial cell surface markers VE-cadherin/CD144 and PECAM1/CD31; Sca-1/Ly6a is highly expressed by arteriolar/arterial endothelial cells but is generally expressed at lower levels by sinusoidal vessels^[4]^, whereas stabilin-2 (Stab2) is generally expressed in sinusoidal vessels but not in the arterial/arteriolar vessels^[6]^. In some studies, IL6st and Dil-labeled acetylated low-density lipoprotein (Dil-Ac-LDL)^[4,7]^ are other markers for sinusoidal bone marrow endothelial cells.

Bone marrow vascular niches harbor HSC in perivascular locations. Different subsets of mesenchymal cells also reside at these sites, contributing to niche functions. Neural-glial antigen 2 (NG2)^+^ cells are mesenchymal cells located predominantly close to the arterioles and transitional zone vessels proximal to the bone^[4]^, whereas the Leptin receptor (LepR)^+^ mesenchymal cells^[8]^ and the Cxcl12-abundant reticular cells^[9]^ are mostly proximal to the sinusoids at the center of the bone cavity. There is evidence to suggest that HSC in these distinct sinusoidal and arteriolar niches are functionally different. However, there is currently no consensus on the relative contributions of distinct vascular niches to the function of these HSC pools. In part, this relates to the incomplete specificity of the lineage-marking tools used to define cell types in the niche and the selection of mouse lines where Cre-mediated recombination occurs in overlapping cell populations with different penetrance^[3,10]^. For example, earlier studies identified Tie2-expressing “arteriolar” endothelium as critical for the maintenance of quiescent HSC^[4]^. However, Tie2 (Tek), the receptor for angiopoietin ligands, is expressed in all bone marrow endothelial subsets^[7]^.

Physical differences in permeability properties of arteriolar and sinusoidal bone marrow endothelia have been proposed to control stemness and migratory capacity of HSC. The more permeable sinusoidal endothelium, as opposed to the less permeable arterioles, would favor HSC transmigration from the bone marrow to the blood and would promote HSC differentiation by allowing exposure to increased plasma levels of reactive oxygen species compromising stemness^[11]^. In this scenario, the major pool of HSC, proximal to the sinusoids, is responsible for the physiological circadian release of HSC from the bone marrow to the blood and for continued replacement of blood cells through differentiation, under the control of sympathetic nerves^[12,13]^. Instead, the smaller pool of quiescent HSC in the arteriolar niche adjacent to the bone would be protected from myelotoxic stress generated by irradiation or chemotherapy, ensuring their availability for hematopoietic regeneration^[14]^.

Different signals have been proposed to contribute the HSC localization at the endosteal site and to promote HSC quiescence. These signals include: Cxcl12 from Cxcl12-abundant reticular cells^[15]^; granulocyte colony-stimulating factor (G-CSF)^[16]^, bone morphogenic protein (BMP)^[17]^, Jag1^[18]^, angiopoietin-1^[19]^, and thrombopoietin from osteoblastic cells^[20]^; extracellular calcium levels and adhesion to the extracellular matrix protein collagen-1^[21]^; and Kitl/SCF from Nestin^+^ mesenchymal stem cells^[22]^ and arteriolar endothelial cells^[4]^. However, the recovery of quiescent HSC near sinusoids questions a clear distinction between HSC in the arteriolar/endosteal and sinusoidal niches^[2]^, suggesting instead the existence of different types of quiescent HSC and other spatially distinct niches/niche factors for maintenance of HSC quiescence. The most quiescent HSC in aged mice reside in sinusoidal niches, which appear critical to protecting HSC from aging; endothelial-derived Jag2 may be an essential sinusoidal niche factor^[23]^. However, single-cell RNAseq results have indicated Jag2 expression in arterial endothelial cells of bone marrow^[7]^. Consistent with the possibility of different types of HSC niches, hematopoietic progenitor cells committed to lymphoid differentiation are found preferentially in the arteriolar niche whereas myeloid-biased progenitors are found in the sinusoidal niche^[24]^. It is interesting that aging is associated with a decline in B-cell production and an increased propensity for myeloid cell production in mice and man^[25,26]^. In addition, Cxcl12-abundant reticular cells, most abundant near sinusoids in the central bone marrow but also present in the endosteal niche^[27]^, can promote HSC quiescence through Cxcl12 secretion and CXCR4 signaling in the HSC^[9,15]^. Non-myelinating Schwan cells that wrap around small axons, which localize in the central and endosteal bone marrow^[27]^, are reported to produce transforming growth factor beta (TGF-β), which activates SMAD-induced quiescence in the adjacent HSC^[28]^. In addition, megakaryocytes, which are broadly distributed in the bone marrow^[29]^, are reported to regulate HSC quiescence through secretion of CXCL4 (also named platelet factor 4)^[29]^, TGF-β^[30]^, and thrombopoietin^[31]^. Thus, niches for quiescent HSC may not be spatially segregated to the periosteal bone marrow. Different quiescent HSC subsets may exist with distinct lineage commitment potential. Quiescent stem cells may be a dynamic and migratory cell pool capable of changing residence in the bone marrow, switching between quiescence/growth/differentiation in response to niche-derived signals. Although quiescence is believed to represent an essential property of HSC to maintain their self-renewal potential in the bone marrow^[14–21,32]^, stem cells in various other tissues do not require quiescence for stemness^[33]^.

Despite these uncertainties, the importance of niche cells and factors for the maintenance of HSC is supported by the substantial reduction of the HSC pool after depleting stem cell factor (SCF/Kitl) from Tie2^+^ endothelial cells or from leptin receptor (LepR)-expressing mesenchymal cells^[7,8,34,35]^ and depleting Cxcl12 from nestin^+^, but not from Lepr^+^ mesenchymal cells^[34]^. In addition, depleting nerve/glial antigen 2 (NG2)^+^ (Cspsg4) mesenchymal cells, but not Lepr^+^ mesenchymal cells, reduced the number of HSC and altered HSC localization in the bone marrow^[34]^.

## CONTRIBUTION OF BONE MARROW NICHES TO HEMATOLOGICAL MALIGNANCIES

**Figure 1.**
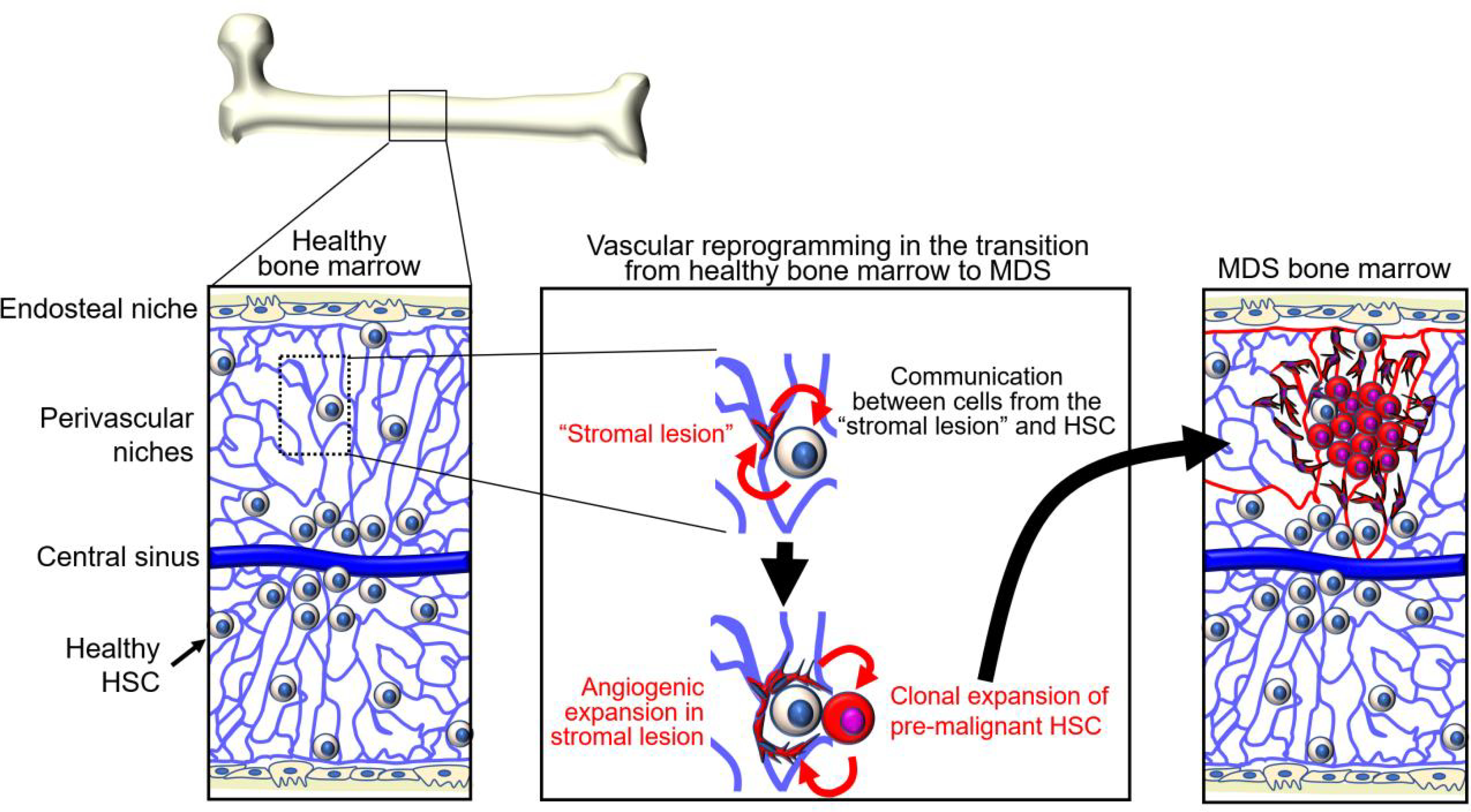
Adaptation of niches and hematopoietic stem cells (HSC) during the transition from healthy bone marrow to myelodysplastic syndromes (MDS). Niches and HSC interdependently generate gene regulatory networks. Niche functions gradually deteriorate with aging, tissue regeneration, and other stress. Changes in niche functions enforce enrichment of HSC subpopulations that have a growth advantage compared to healthy HSC. Pre-malignant and malignant cells remodel the niches. Bi-directional alterations are soil and seed toward MDS.

Appreciation of the importance of niche cells and factors in physiologic hematopoiesis suggested that “derangements” in bone marrow “stroma” may contribute to clonal selection of bone marrow malignancies^[36]^ [Figure 1]. Neoplastic hematopoietic cells could induce change in the bone marrow stroma or “morph into” stroma resulting in improved growth conditions for the tumor cells. Alternatively, stromal lesions could be the inducers of malignant transformation of hematopoietic cells^[36–38]^.

Experimental mouse models have explored the possibility that non-hematopoietic niche factors contribute to the initiation of bone marrow malignancy. Proof-of-principle came from genetic disruption of *Dicer1* ribonuclease in osterix^+^ osteo-lineage progenitors. The mutant mice displayed impaired osteoblast differentiation and myelodysplasia associated with leukopenia when the mice were 4–6 weeks old. A proportion of these mice developed acute myeloid leukemia (AML) and rapidly died^[39]^. *Dicer1* was not disrupted in the leukemic cells, which instead displayed other genetic abnormalities, providing evidence that Dicer1 depletion from osteo-lineage cells caused myelodysplasia and AML in mice. The Schwachman-Bodian-Diamond syndrome (*Sbds*) gene was expressed at significantly lower levels in the osteo-lineage cells after *Dicer1* disruption, and deletion of *Sbds* in osterix^+^ osteoprogenitors produced leukopenia, lymphopenia, and myelodysplasia, recapitulating key features of Dicer1 deficiency in the same cells^[39]^. This is important because the *Sbds* gene is mutated in Schwachman-Bodian-Diamond syndrome, a rare, inherited form of bone marrow failure, characterized by leukopenia and a significantly increased chance of developing MDS and AML. However, *Sbds* gene knockdown in hematopoietic progenitors only caused neutropenia but did not fully recapitulate the clinical syndrome, supporting a role of non-hematopoietic bone marrow cells in the full development of the syndrome^[39]^.

Consistent with these observations in mice, mesenchymal stromal cells from patients with MDS showed abnormally low levels of *Dicer1*, *Drosha*, and *Sbds* mRNA expression compared to controls^[40]^. Mechanistic studies in *Sbds*-deficient mice that develop myelodysplasia found evidence of oxidative stress and activation of the DNA damage response in the HSC and progenitors following perturbation of the mesenchymal compartment. This genotoxic response in HSC and progenitors was linked to activation to a TLR4 pattern recognition receptor/S100A8/9 stress response^[41]^. These studies pointed to a role of both hematopoietic and niche defects as potentially contributing to the development of Schwachman-Bodian-Diamond syndrome.

Other genetic experiments altering niche components support a role of the microenvironment in the development of MDS or MDS-related MPN, which carry a high risk for leukemia development^[42]^. For example, mice expressing a constitutively active β-catenin in pro-α1(I)collagen^+^ in osteoblasts develop anemia by two weeks of age associated with multilineage bone marrow dysplasia followed by the development of AML and death by 6 weeks of age. Transplantation of bone marrow cells from these leukemic mice into lethally irradiated wild type recipients resulted in leukemia and early death^[43]^. In related experiments, the loss of a single allele of APC, an inhibitor of the Wnt/β-catenin pathway, altered the function of HSC and hematopoietic progenitors, leading to MDS-like disease in the mouse^[44,45]^. These mouse models may be relevant to human MDS with del(5q) in that the *APC* gene, located on 5q23, is deleted in more than 95% of cases of (5q) MDS^[46]^.

A series of other experiments provided evidence of premalignant hematopoietic disorders initiated by non-hematopoietic cells. In one example, newborn mice lacking NF-κB inhibitor-α (*IκBα/Nfkbia*) developed myeloproliferative disease affecting the granulocyte, erythroid monocyte/macrophage lineages. This outcome could not be reproduced by the selective conditional deletion of IκBα in myeloid lineage cells. By contrast, coculture of IκBα deficient hepatocytes with wild type bone marrow cells induced an increase of multilineage (granulocyte/erythroid/monocyte/macrophage) colonies^[47]^. In another example, the conditional deletion of the retinoblastoma protein (*Rb1B*) in the hematopoietic system produced a myeloproliferative syndrome with loss of HSC. This phenotype was not attributable to *Rb1B* deficiency in the HSC, as it could not be reproduced by deleting *Rb1B* from the bone marrow HSC, but instead to *Rb1B* deficiency in the bone marrow microenvironment as demonstrated in transplant experiments^[48]^. Also, inactivation of Mind bomb-1 (Mib1), a protein that regulates Notch signaling by facilitating the internalization of Notch ligands, induced a myeloproliferative disorder with progressive tissue accumulation of immature granulocytes and death of the mice by approximately 20 weeks. Transplantation of normal bone marrow into the *Mib1*-null mice reproduced the myeloproliferative syndrome in the transplanted graft, implicating the recipient bone marrow microenvironment^[49]^. Furthermore, mice with Crebbp (CREB-binding protein) haploinsufficiency, display increased myeloid cell differentiation, loss of HSC and hematopoietic progenitors, and are prone to developing hematological malignancies with age. These abnormalities correlated with increased expression of the endothelial adhesion molecule 1 and CDH5/VE-cadherin in a subset of bone marrow endothelial cells from the *Crebbp*^+/−^ mice^[50]^. In addition, mice deficient of retinoic acid receptor gamma (*Rarg*) display progressive expansion of granulocyte/macrophage progenitors and mature granulocytes in the bone marrow and blood. Since bone marrow from normal mice developed a similar myeloproliferative disorder after transplantation into *Rarg*-deficient mice, intrinsic deficiency of *Rarg* in the hematopoietic cells is unlikely the cause of the myeloproliferative syndrome^[51]^. Another example linked activating mutations of *Ptpn11* in the mouse bone marrow mesenchymal stem cells, but not within the osteoblasts or endothelial cells, to the development of and progression of myeloproliferative disease^[52]^. *Ptpn11* codes for the Src homology region 2 domain-containing phosphatase 2 (SHP2). Mechanistically, the process was attributed to a pro-inflammatory bone marrow milieu sustained by interleukin-1β^[52]^. This mouse model could be relevant to a proportion of patients with juvenile myelomonocytic leukemia arising either in patients with Noonan syndrome, an autosomal dominant disorder characterized by skeletal and heart defects, or without Noonan syndrome. Noonan syndrome is attributed to germline mutations of *Ptpn11* that affect the SHP2 phosphatase domains^[53]^. In addition, one third of patients with juvenile myelomonocytic leukemia without the syndrome harbor somatic mutations of *Ptpn11*, which were predicted to cause a gain of function in SHP2 that was proposed to act as an oncoprotein in these myeloid leukemias^[54]^.

## MALIGNANT CELLS REMODEL THE BONE MARROW NICHE

Evidence that niche pathology drives bone marrow hematological malignancies comes primarily from genetic experiments in mice outlined in the previous section, whereas clinical evidence for this process is currently limited. Only rare cases of MDS and AML displayed genetic mutations in bone marrow mesenchymal cells, which differed from those detected in the malignant cells, supporting an oncogenic role of non-hematopoietic niche factors^[55–58]^. However, these mutations were of unknown oncogenic potential. Additional support for a primary pro-oncogenic role of the bone marrow microenvironment comes from a small series of leukemias arising in allogeneic transplant recipients, where some of the leukemias were of normal donor derivation and may thus have arisen because of their localization in the recipient pathogenic bone marrow^[59]^.

There is instead considerable evidence that the bone marrow microenvironment is altered when malignant cells are present, and that malignant cells can drive change in the bone marrow microenvironments in which they reside. These changes involve primarily the mesenchymal and endothelial cell components of the niche.

### Mesenchymal cells

Compared to age-matched healthy controls, mesenchymal stem cells (MSCs; defined as bone marrow cells capable of differentiating into cartilage, bone, and fat) derived from MDS or AML bone marrows display a reduced proliferative capacity, altered cell surface marker profile and disrupted secretory function, particularly for osteopontin, Kit-ligand (Kitl/SCF), vascular endothelial growth factor-A (VEGF), placental-derived growth factor (PlGF/PGF), angiopoietins, inflammatory cytokines (including IL-1β, IL-6, and TNFα), and chemokines (including CXCL12 and CCL3); these alterations in MSCs, typically associated with senescence, have been implicated in increased malignant cell survival and resistance to immune recognition and chemotherapy^[57,60–67]^. Clonal hematopoiesis associated with aging is often associated with a pro-inflammatory bone marrow stroma, in part because the same mutations that cause clonal expansion of hematopoietic cells also lead to increased expression of pro-inflammatory genes^[37]^.

In AML, bone marrow “stroma” is focally depleted, particularly in endosteal areas with high infiltration of tumor cells, suggesting that AML cells remodel the stroma when present in sufficient number^[68]^. Progressive depletion of bone marrow stroma is associated with a progressive loss of healthy HSC and osteoblastic cells^[68]^. Consistent with these observations *in vivo*, MDS-derived MSCs display a reduced capacity to support the survival of co-cultured normal CD34^+^ HSC. This defect was linked to epigenetic and transcriptional changes in MDS-derived MSCs^[66]^. The addition of a hypomethylating agent to co-cultures of normal CD34^+^ cells and MDS-derived MSCs corrected the CD34^+^ cell deficiency^[66]^. MSC-derived exosomes may mediate the communication of MDS-MSC with normal HSC^[69]^. In turn, there is evidence that healthy MSCs adopt phenotypic characteristics of MDS-derived MSCs when exposed to hematopoietic cells from MDS patients, providing evidence that MDS hematopoietic cells display a pathogenic instructive phenotype^[65,67]^.

An example of a pathogenic axis linking AML cells with bone marrow stromal cells involves the tyrosine kinase Axl receptor. In this example, AML cells prompt secretion of Gas6 (growth arrest-specific 6) protein from stromal cells. Gas6 promotes proliferation, survival and chemoresistance of Axl^+^ AML cells^[70]^. Secreted factors, exosomes and vesicular particles derived from malignant hematopoietic cells were found to be mediators of MSCs remodeling in the malignant bone marrow. Extracellular vesicles derived from malignant AML clones were reported to contribute to re-shaping the niche into a leukemia-permissive microenvironment by inhibiting production of HSC-supportive factors^[71,72]^. AML-derived extracellular vesicles were also found to transmit endoplasmic reticulum stress to the bone marrow stroma and to promote MSC differentiation into osteo-lineage cells through bone morphogenic protein 2^[73]^. In addition, tunneling nanotubules could facilitate direct transfer of mitochondria from bone marrow stromal cells to the leukemic cells^[74]^. An example of three-way crosstalk between malignant hematopoietic cells, sympathetic nerves, and stromal cells links AML-induced disruption of sympathetic nerves and secondary effects on bone marrow mesenchymal cells expressing beta2 adrenergic receptors (β_2_ adrenoreceptor)^[64,75]^. Thus, it seems that the malignant cells in the bone marrow drive change in the mesenchymal cell microenvironment, and the affected mesenchymal cells can, in turn, propagate change in the normal bone marrow mesenchymal cells, altering niche support functions for the normal bone marrow HSC, and promoting growth in the malignant cells.

### Vascular reprogramming

The density of blood vessels is markedly increased in MDS, *de novo* AML, and MPN compared to the normal bone marrow^[76–80]^. Morphologically, these vessels are altered showing increased tortuosity, increased permeability, and focal vascular damage with loss of the endothelial monolayer, all of which are vascular features commonly observed in solid tumors^[68,81,82]^. In mouse models of AML, vascular abnormalities are associated with poor vessel perfusion and bone marrow hypoxia, particularly surrounding the tumor cells, and these deficiencies are thought to limit proper delivery of anti-cancer drugs^[68,82]^. AML cells and other cell types in the leukemic bone marrow secrete VEGF (also identified as vascular permeability factor, VPF)^[83]^. Hypoxia is a principal regulator of VEGF expression through stabilization of the hypoxia-inducible factors HIF-1α and HIF-2α. Increased oxygen consumption by rapidly proliferating tumor cells contributes to bone marrow hypoxia, and increased VEGF expression from different cell sources in the bone marrow microenvironment likely contributes to increased vascularization and vascular permeability in the leukemic bone marrow^[84]^. Circulating levels of the pro-angiogenic factors VEGF and angiopoietin-2 are abnormally increased in mice with AML and other leukemias^[68,83,85]^. In addition, increased systemic levels of the endothelial growth factors VEGF, FGF2, and hepatocyte growth factor (HGF) directly correlated with increased bone marrow vascularization in MDS, AML, and MPN^[76,77,79,81]^. However, the contribution of tumor hypoxia to bone marrow leukemogenesis is currently unclear since different studies show contrasting results in different mouse models of AML^[86–88]^. Certain leukemic cells express functional VEGFR2 and respond to VEGF with increased proliferation^[83,89]^. VEGF/VEGFR2-activated endothelial cells and potentially other responsive cells in the bone marrow microenvironment can accelerate AML progression, through increased adhesion to AML cells and secretion of cytokines and growth factors that promote leukemic cell growth and viability, including Kitl/SCF, granulocyte-macrophage colony stimulating factor (GMCSF), granulocyte colony-stimulating factor (G-CSF), and IL-6^[82,90,91]^. RNA profiling of endothelial cells retrieved from the bone marrow of mice with AML detected evidence of increased expression of adhesion molecules, particularly integrins associated with activation of Fak signaling^[82]^. One of the most expressed genes was Nox4, a NADPH oxidase involved in the response to hypoxia leading to the production of reactive oxygen species, activation of nitric oxide synthase 3, and release of nitric oxide (NO)^[82]^. Consistent with these experimental models, NO was abnormally elevated in bone marrow aspirates of patients with AML^[82]^. Thus, the bone marrow with MDS, AML and MPN is more vascularized than normal bone marrow, but the vessels are morphologically and functionally altered as they are leaky and poorly perfused [Figure 1]. However, we do not know if the process is diffused throughout the bone marrow or limited to specific areas and vascular beds. VEGF constitutively produced by the tumor cells or induced by hypoxia in tumor cells or non-malignant bone marrow cells may be the driver of angiogenesis, as observed in many solid tumor types. Other proangiogenic factors and inflammatory cytokines likely contribute to altering bone marrow vessels and their function, but this remains an area of investigation.

### Tumor-promoting effects of the niche and therapeutic targeting

Current approaches to treatment of patients with MDS evaluate the risk of AML development through a variety of prognostic scoring systems. MDS is most prevalent in the elderly. Lower-risk patients die more frequently from complications of bone marrow failure than from transformation into AML. Consequently, the goal of treatment in these lower-risk patients is symptom relief rather than cure or complete remission. The only curative treatment for MDS with a high or very high risk of transformation into AML is allogeneic bone marrow transplantation. Choice of this treatment is based on assessing medical fitness and on other criteria. No drug intended specifically to target niche deficiencies is currently approved for the treatment of MDS/AML. However, lenalinomide, which is FDA approved for lower-risk MDS and is particularly effective at reducing transfusion requirements in MDS with del(5q), has immunomodulatory and anti-angiogenic functions^[92]^. Azacytidine and decitabine, pyrimidine nucleoside analogs that inhibit DNA methylation and have direct cytotoxicity for abnormal bone marrow hematopoietic cells, may also target the abnormal mesenchymal cells, as indicated by co-culture of normal HSC with MDS-derived mesenchymal cells with azacytidine^[66]^. Luspatercept, FDA approved for the treatment of anemia associated with MDS with ring sideroblasts with or without thrombocytosis, traps ligands in the TGF-β superfamily and promotes erythroid differentiation. TGF-β is a bone marrow niche factor produced by many cell types in an inactive form and is then activated in the bone marrow by non-myelinating Schwann cells that coat sympathetic nerves^[28]^. Immunosuppressive therapies with anti-thymocyte globulin and cyclosporine are effective in hypoplastic MDS and/or patients with paroxysmal nocturnal hemoglobinuria-positive cells, rather than directly affecting the malignant cells.

Increased appreciation of bone marrow niches and their contributions to the development and progression of MDS and AML, has brought into focus the niche as a potential therapeutic target. This could be accomplished by blocking interactions between the malignant cells and their niche; targeting the pro-oncogenic bone marrow microenvironment; or targeting the neoplastic cells-derived processes that reprogram the niche(s). One approach has been to remove malignant hematopoietic cells from their supportive niche through mobilization of AML cells using the CXCR4 blocker plerixafor (as done to mobilize normal stem/progenitor cells for hematopoietic transplantation in conjunction with G-CSF). This approach had shown limited benefit in initial trials^[93,94]^, but more potent CXCR4 inhibitors hold promise for treatment of AML^[95]^. CD44/E-selectin is a cell surface glycoprotein receptor for hyaluronic acid, osteopontin, and other ligands involved in adhesion of HSC and leukemic cells. CD44/E-selectin antagonists were effective in experimental mouse models of AML and are now tested in initial clinical trials^[96]^. Similarly, studies *in vitro* showed that VCAM1 (on the stromal cells) interacting with VLA-4 on the leukemic cells induced reciprocal activation of the NF-κB pathway, proposed to promote resistance to chemotherapy^[97]^. The blockade of NF-κB activation reduced drug resistance in AML cells^[97]^. Currently, the VLA4 inhibitor AS101 is being tested in patients with MDS and AML (NCT01010373).

The role of Notch signaling in AML appears complex. Human AML samples display increased expression of Notch receptors, but they are generally inactive or display basal levels of activity. Activation of Notch signaling initiated growth arrest and differentiation of AML cells, suggesting a tumor-suppressive function, which provides a rationale for using Notch agonists for the treatment of AML^[98]^. This approach has not been pursued in clinical development. However, Notch-blocking antibodies neutralized the growth-promoting effects imparted by bone marrow stromal cells, suggesting an oncogenic role of Notch in AML^[99]^. This approach is currently under evaluation in clinical trials in T-ALL/T-LBL (NCT02518113).

Another approach is based on transcriptome profiling of patient-derived AML xenografts, which detected abnormal elevations of endothelial NO in the xenografts and patient samples^[82]^. Experimental evidence additionally showed that NO inhibitors improved the effectiveness of chemotherapy, presumably through vessel normalization and reduced permeability^[82]^. Nitric oxide synthase inhibitors are in clinical trials and have been proposed for clinical testing in AML^[100]^.

Other approaches have focused on reducing bone marrow vascularity that accompanies AML. Vessel density, abnormally high in MDS and AML, is directly correlated with poor patient outcome. After initial encouraging results^[101]^, a randomized phase-2 trial with the VEGF inhibitor, avastin, did not improve the therapeutic outcome of patients with AML^[102]^. A different approach to reducing the bone marrow vasculature has focused on blocking Tie2/angiopoietin interaction. The Tie2 receptor is generally expressed by AML cells, whereas its ligands angiopoietin-1 and −2 ligands are expressed at abnormally high levels in AML bone marrow^[103]^. AMG386, a peptibody that blocks TIE2-angiopoietin 1/2 binding, has undergone a successful initial trial in AML patients^[104]^ and is currently under investigation in combination with AraC (NCT01555268).

Pre-clinical/clinical development of drugs and strategies for targeting the bone marrow niche in hematological malignancies is in its early stages. The potential for success of this approach is supported by the evidence that the niche is a critical component of pathogenesis in MDS, AML, and other hematological malignancies and contributes to therapy resistance. The timing of intervention with niche targeted therapies may be important. Observations from AML transplants into irradiated mice suggest that AML sensitivity to niche-derived signals decreases as the disease advances. In one such example, AML cells from early-stage disease homed to the endosteal bone marrow and were responsive to Wnt signaling, whereas cells from more advanced AML homed more centrally in the bone marrow and were less sensitive to Wnt signals^[105]^.

## CONCLUSION

Research over the past few years has revealed the importance of complex interactions linking the bone marrow microenvironment with healthy HSC and their malignant counterparts. Initial insight into the bone marrow niche has evolved to recognize specific cell partners, avenues of cell communication and biochemical pathways underlying flexible and specific biological outcomes. Some potentially critical contributors to malignant hematopoietic cell growth have emerged, others are not fully verified, and new ones will probably be identified. Nonetheless, current knowledge provides opportunities for therapeutic exploitation of targets already identified as effective to prevent and mitigate the growth of malignant hematopoietic clones. Either new drugs or more potent versions of current drugs could be effective against these targets. Recent discoveries also provide opportunities for the identification of new drug targets to alleviate pro-oncogenic pathways originating from the malignant niche. Drug discovery targeting the niche and clinical trials to evaluate their safety and effectiveness hold great promise for developing novel therapeutics for hematological malignancies.

## Figures and Tables

**Table 1.** Markers of bone marrow blood vessels

Vascular markers	Artery	Arteriole	Sinusoid

PECAM1/CD31	+	+	+
VE-cadherin/CD144	+	+	+
Sca-1/Ly6a	+	+	low
Stabilin-2/Stab2	−	−	+
IL6st	−	−	+
Dil-Ac-LDL	−	−	+
α-SMA	+	−	−
